# Not Bad: Passive Leg Raising in Cardiopulmonary Resuscitation-A New Modeling Study

**DOI:** 10.3389/fphys.2016.00665

**Published:** 2017-01-09

**Authors:** Yanru Zhang, María Jiménez-Herrera, Christer Axelsson, Yunzhang Cheng

**Affiliations:** ^1^School of Medical Instrument and Food Engineering, University of Shanghai for Science and TechnologyShanghai, China; ^2^Nursing Department, Universitat Rovira I VirgiliTarragona, Spain; ^3^Faculty of Caring Science, Working Life and Social Welfare, University of Borås, The Prehospital Research Centre of Western GötalandBorås, Sweden

**Keywords:** cardiopulmonary resuscitation, passive leg raising, thoracic/cardiac pump effect, coronary perfusion, cerebral perfusion

## Abstract

**Aim:** To evaluate, using a simulated haemodynamic circulation model, whether passive leg raising (PLR) is able to improve the effect during cardiopulmonary resuscitation (CPR); to expose the possible reasons why PLR works or not.

**Materials and Methods:** We adapted a circulatory model for CPR with PLR. First we compared cardiac output (CO), coronary perfusion pressure (CPP), blood flow to heart (Q_heart_), and blood flow to neck and brain (Q_head_) of standard chest compression-only CPR with and without PLR; second we simulated the effects of PLR in different situations, by varying the thoracic pump factor (TPF) from 0 to 1; third we simulated the effects when the legs are lifted to the different heights. Finally, we compared our results with those obtained from a published clinical study.

**Results:** According to the simulation model, (1) When TPF is in the interval (0,1), CPP, CO, Q_heart_, and Q_head_ are improved with PLR, among them with half-thoracic/half-cardiac pump effect (TPF is 0.5), CPP, CO, Q_head_, and Q_heart_ increase the most (by 14, 14, 15, and 17%). (2) When TPF is 1 (pure thoracic pump, with an emphysema or extremely thick thorax), PLR has almost no effect on CPP, CO, and Q_heart_ (−1, 2, and 0%), whereas Q_head_ is increased by 9%; (3) Regardless of whether there is a cardiac or thoracic pump effect, PLR is able to increase Q_head_ by 9–15%. (4) When the legs are lifted to 30° to the ground, the volume transferred from legs to upper body is 36% of the initial volume in legs; when the legs are lifted to 45°, the volume transferred is 43%; when the legs are lifted to 60°, the volume transferred is 47%; when the legs are lifted to 90°, the volume transferred is 50%.

**Conclusion:** Generally PLR is able to achieve improved cerebral perfusion and coronary perfusion. In some extreme situations, it has no effect on cardiac output and coronary perfusion, but still improves cerebral perfusion. PLR could be a beneficial supplement to CPR, and it is not necessary to lift the legs too high above the ground.

## Introduction

Cardiac arrest (CA) is a sudden stop in effective blood circulation due to the failure of heart contraction (Jameson et al., 2005). It is a major health issue affecting a large population in Europe and the United States (Rea et al., 2004; Atwood et al., 2005). Each year in the United States, 330,000 people suffer a cardiac arrest and only 8% of them survive—leaving 300,000 fatalities Why Learn Cpr and Aed Use? 2014. In Asia, the survival rate is even lower (Berdowski et al., 2010). According to cardiopulmonary resuscitation (CPR) guidelines, the treatment for cardiac arrest is immediate chest compressions, rescue breathing, and early electrical defibrillation (Cummins et al., 1991). Clinicians and researchers have used every means available to achieve a rapid and effective rise in coronary perfusion pressure (CPP) (Ralston et al., 1982; Cohen et al., 1992; Tang et al., 1997; Yuan et al., 2007). However, the return to spontaneous circulation (ROSC) rate remains low and more than half of the survivors have different degrees of brain injury (Pusswald et al., 2000; Laver et al., 2004; Nolan et al., 2008; Oddo and Rossetti, 2011).

Passive leg raising (PLR) is a maneuver which involves the elevation of the lower limbs from the horizontal plane (Dragoumanos et al., 2012). The effect of PLR is to shift blood from the lower extremities toward the intra-thoracic compartment (Pottecher et al., 2010). As it is a simple and effective maneuver, it has recently attracted increasing interest, like various tests for monitoring heart function, assessing fluid responsiveness (Teboul and Monnet, 2008; Preau et al., 2010; Pinsky, 2015) and unmasking pulmonary hypertension (Girerd et al., 1989; Ohashi et al., 1997). However, before its popularity in fluid responsiveness tests, it was a rescue maneuver that had been used for years by first-aid rescuers and it was recommended as part of CPR in international recommendations before 1992 (Standards and guidelines for cardiopulmonary resuscitation (cpr) and emergency cardiac care (ecc), 1974, 1980; Standards and guidelines for cardiopulmonary resuscitation (cpr) and emergency cardiac care (ecc). National academy of sciences - national research council, 1986). In 1992, it was removed from the guidelines (Guidelines for cardiopulmonary resuscitation and emergency cardiac care. Emergency cardiac care committee and subcommittees, american heart association. Part ii. Adult basic life support, 1992) as there was a lack of clinical evidence to support its effectiveness. In recent years, the discussion related to PLR in CPR has been re-opened by clinicians and researchers. Axelsson et al. found PLR can increase end-tidal carbon dioxide (PETCO2) during CPR (Axelsson et al., 2010); The on-going study by Jiménez-Herrera et al. is investigating whether PLR can improve the 1-month survival rate of the patients who had out-of-hospital cardiac arrest (OHCA) (Jimenez-Herrera et al., 2014); Dragoumanos et al. found that PLR during CPR produced significantly higher neurological scores in piglets (Dragoumanos et al., 2012).

A clinical study has shown that bystander CPR before emergency medical service (EMS) arrival improved 30-day survival rate compared to no CPR before EMS arrival (Hasselqvist-Ax et al., 2015). Passive leg raising is a simple and fast maneuver, it can be easily applied by bystanders. If applying PLR would improve the effect of CPR, it is possible to further increase the survival rate of CA patients. However, to date, we still do not have strong clinical evidence or consensus that PLR can improve the effect of CPR. Therefore, in this study we aim to assess the effect of PLR with a modeling study. We compared CPP, cardiac output (CO), blood flow to the heart, and brain before and after PLR; we explored the impact of individual difference on the effect of PLR; and we studied the effects of lifting the legs to different heights.

## Materials and methods

### *In silico* model description

**Figure 1:**
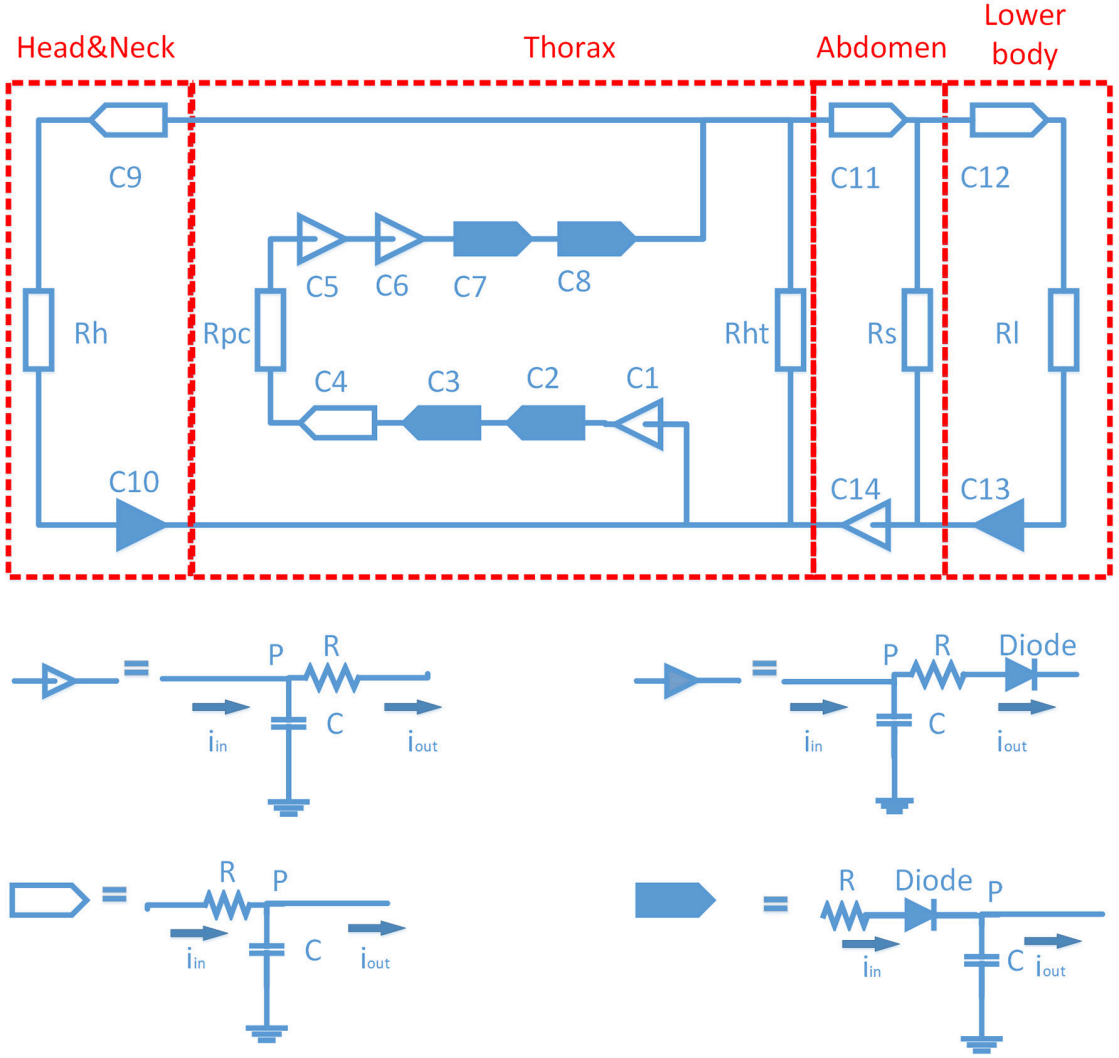
**Diagram of circulation model**. There are 14 compartments (C1-C14). “
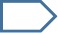
” stands for artery or ventricle compartment; “
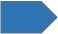
” stands for artery or ventricle compartment with a valve; “
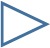
” stands for vein or atrium compartment, “
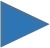
” stands for vein or atrium compartment with a valve.

The computer model used in this study was essentially based on the circulatory model by Babbs (2005). We modified it to simulate PLR. It is a fourteen-compartment close-loop model (C1-C14, Figure 1). It includes four heart chambers, the pulmonary circulation, the thoracic aorta feeding an upper body compartment (head and neck) and an abdominal compartment, the latter feeds the lower body (legs, buttocks) compartment. The structure of each lumped artery and vein compartment includes a resistance (R) followed by a capacitance (C). The peripheral circulation for each compartment is a simple resistance. The valves are simulated by diodes, to ensure unidirectional blood flow. In view of the low blood velocity in CPR, inertial effects were neglected. The definitions and values of the compartments are summarized in Table 1, in which we also give the initial volumes in all compartments. The whole blood volume is 4.36 L, the subject starts in the condition of cardiac arrest, where the blood volume is distributed over the various compartments. More detailed description in this model can be found in references (Babbs, 2005; Zhang and Karemaker, 2012).

**Table 1:** **Definitions and values of each compartment in the model**.

**Symbols**	**Definitions**	**Symbol**	**R:mmHg/L/s C:L/mmHg**	**Initial volume (L)**
C1	Right atrium and intrathoracic great veins	R	0	0.367
		C	0.00950	
C2	Right ventricle	R	5	0.150
		C	0.016	
C3	Large pulmonary arteries	R	10	0.109
		C	0.0042	
C4	Peripheral pulmonary arteries	R	10	0.160
		C	0.00042	
C5	Peripheral pulmonary veins	R	5	0.300
		C	0.00128	
C6	Central pulmonary veins and left atrium	R	0	0.250
		C	0.0128	
C7	Left ventricle	R	5	0.150
		C	0.0080	
C8	Thoracic aorta	R	10	0.078
		C	0.00080	
C9	Carotid arteries	R	60	0.132
		C	0.00020	
C10	Jugular veins	R	30	0.216
		C	0.01200	
C11	Abdominal aorta	R	25	0.114
		C	0.00040	
C12	Femoral arteries	R	360	0.133
		C	0.00020	
C13	Femoral veins	R	180	0.267
		C	0.00470	
C14	Inferior vena cava	R	25	1.930
		C	0.02340	
Rh	Resistance of head vasculature		5520	N/A
Rht	Resistance of coronary vessels		10780^*^	
Rs	Resistance of splanchnic vasculature		1800	
Rl	Resistance of leg vasculature		8520	
Rpc	Resistance of pulmonary capillary bed		105	

* *In compression phase, Rht is considered “infinite,” there is no coronary flow; in restoring phase, Rht is 10780 mmHg/L/s*.

Chest-compression-only CPR (CO-CPR) was modeled by applying external forces to the compartments (C1–C8) in the chest chamber. Chest compression simultaneously increased the intrathoracic (P_lung_) and mediastinal pressure (P_M_), where the intrathoracic pressure worked on all the compartments in the chest, while the mediastinal pressure led to compression of the heart and/or the rest of the compartments in the chest, depending on the value of thoracic pump factor [TPF: (0–1)].

The thoracic pump factor [TPF: (0–1)] is the degree to which the “thoracic/cardiac pump” mechanism of CPR works. When TPF is 0, it refers to pure cardiac pump, such as in the case of open chest or an extremely thin thorax (Torres and White, 1997), the mediastinal pressure worked only on the ventricles (C2, C7); when TPF is 1, it refers to pure thoracic pump, such as in the case of emphysema or an extremely thick and deep thorax, the mediastinal pressure worked on all the compartments in the chest, except the vasculatures deeply hidden in the lungs (peripheral pulmonary arteries and veins: C4, C5).

We ran the model by different TPF values (0, 0.25, 0.5, 0.75, and 1) to stand for different situations. The formulas that involve TPF are listed below. “*P*” stands for pressure, “*V*” stands for volume, “*I*” stands for blood flow, “*f*_*tp*_” stands for TPF, and subscripts indicate which compartment the formulas describe.

(1) $$\begin{array}{l} \{\begin{array}{l} \Delta V_{C1}=(i_{in_{C1}}-i_{out_{C1}})\Delta t=[\max\text{​}\left(0,\frac{P_{C10}-P_{C1}}{R_{C10}}\right)\\ \;+\frac{P_{C14}-P_{C1}}{R_{C14}}-\max\left(0,\frac{P_{C1}-P_{C2}}{R_{C1}}\right)]\text{​}\Delta t\\ \Delta P_{C1}=\frac{\Delta V_{C1}}{C_{C1}}+\Delta P_{lung}+f_{tp}\Delta P_{M} \end{array} \end{array}$$

(2) $$\begin{array}{l} \{\begin{array}{l} \Delta V_{C3}=\left(i_{in_{C3}}-i_{out_{C3}}\right)\text{​}\Delta t=[\max\text{​}\left(0,\frac{P_{C2}-P_{C3}}{R_{C2}}\right)\\ \;-\;\frac{P_{C3}-P_{C4}}{R_{C3}}]\Delta t\\ \Delta P_{C3}=\frac{\Delta V_{C3}}{C_{C3}}+\Delta P_{lung}+f_{tp}\Delta P_{M} \end{array} \end{array}$$

(3) $$\begin{array}{l} \{\begin{array}{l} \Delta V_{C6}=\left(i_{in_{C6}}-i_{out_{C7}}\right)\text{​}\Delta t=[\frac{P_{C5}-P_{C6}}{R_{C5}}\\ \;-\max\text{​}\left(0,\frac{P_{C6}-P_{C7}}{R_{C6}}\right)]\Delta t\\ \Delta P_{C6}=\frac{\Delta V_{C6}}{C_{C6}}+\Delta P_{lung}+f_{tp}\Delta P_{M} \end{array} \end{array}$$

(4) $$\begin{array}{l} \{\begin{array}{l} \Delta V_{C8}=\left(i_{in_{C8}}-i_{out_{C8}}\right)\text{​}\Delta t=[\max\text{​}\left(0,\frac{P_{C7}-P_{C8}}{R_{C7}}\right)\\ \;-\;\frac{P_{C8}-P_{C9}}{R_{C8}}]\Delta t\\ \Delta P_{C8}=\frac{\Delta V_{C8}}{C_{C8}}+\Delta P_{lung}+f_{tp}\Delta P_{M} \end{array} \end{array}$$

### CO-CPR and PLR maneuver simulation

We simulated CO-CPR, as applied by one rescuer (as control group), and CO-CPR with PLR, by two rescuers together.

In keeping with the new guidelines, a compression frequency of 100/min was used; no time was devoted to ventilation. The chest was compressed with a force of 400 N, or around 40 kilograms, which clinically related to a depth of 5.1 cm; non-overlapping half-sinusoids with a 50% duty cycle were used (Guyton et al., 1954; Babbs, 2005) as external pressure waveforms.

Passive leg raising induced a gravitational transfer of blood volume from the lower body toward the central circulation. We assumed a homogeneous pressure in the lower body cavity. The model simulated an average man: height 175 cm, weight 70 kg, leg length 79 cm (45% of height) (Frederick et al., 2010; Metropolitan life insurance company tables men, 2016). In Figure 1 the legs were lumped into one arterial compartment, connected with one venous compartment, which allowed the hypothesis that the volume in arteries was a uniform cylinder, and so was the volume in veins. The cross-section area could be calculated by initial volume divided by length of legs (79 cm long). We assumed that the vessels will not dilate or collapse during PLR.

**Figure 2:**
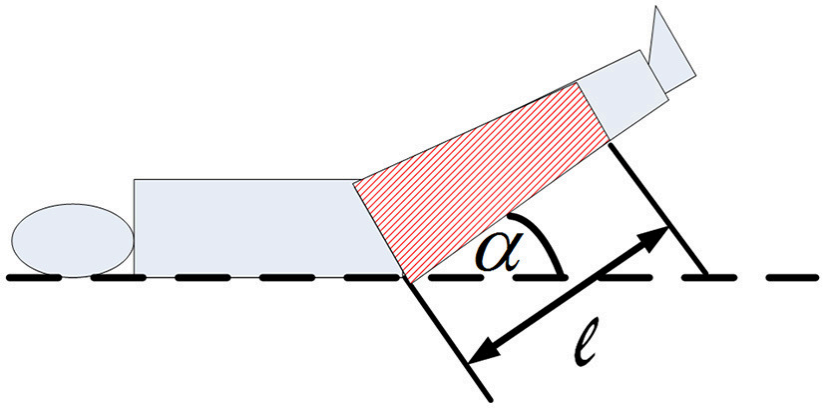
**Diagram of passive leg raising**. α: the angle between the horizontal plane and raised legs, *l*: the length of the blood volume in legs.

When the legs were lifted to different heights (consequently different angles between the horizontal plane and the legs), the gravitational effect is shown in Figure 2, Equation (5):

(5) $$\begin{array}{l} {\text{P}}_{PLR}=\text{ρgh}=\text{ρg}\cdot \frac{1}{2}\cdot \text{l}\cdot \text{sinα} \end{array}$$

Where P_*PLR*_ is the pressure (Unit is Pa), ρ is the density of blood (1060 kg/m3), g is the gravitational acceleration (9.8 N/kg), and l is the height of the volume in legs and α is the angle between the horizontal plane and the lifted legs. Since the volume is distributed uniformly the center of gravity is at the medium point of the volume in legs.

Given the above settings, the impacts of PLR on the circulation can then be simulated by connecting the femoral (arteries and veins) capacitances to a positive potential point instead of zero potential point (the ground). The blood flow through the femoral arteries and veins are shown in Equation (6, 7).

(6) $$\begin{array}{l} \{\begin{array}{l} \Delta V_{C12}=(i_{in_{C12}}-i_{out_{C12}})\Delta t\\ =[\frac{P_{C11}-P_{C12}}{R_{C12}}-\frac{P_{C12}-P_{C13}}{R_{l}}]\text{​}\Delta t\\ \Delta P_{C12}=\frac{\Delta V_{C12}}{C_{C12}}+\Delta P_{PLR} \end{array} \end{array}$$

(7) $$\begin{array}{l} \{\begin{array}{l} \Delta V_{C13}=\left(i_{in_{C13}}-i_{out_{C13}}\right)\text{​ }\Delta t\\ =[\frac{P_{C12}-P_{C13}}{R_{l}}-\text{max}\left(0,\frac{P_{C13}-P_{C14}}{R_{C13}}\right)]\text{​ }\Delta t\\ \Delta P_{C13}=\frac{\Delta V_{C13}}{C_{C13}}+\Delta P_{PLR} \end{array} \end{array}$$

In this study we also studied the effects of lifting the legs to different heights through simulation (α equals to 30, 45, 60, 90°).

The model was solved using MAT LAB/Simulink. The solver was ODE4. To make the model reach steady state in a convenient time frame, step size was set at 0.001 s; simulation time was 40 s. The model reached a steady state after 8 s.

### Referenced clinical study

We compared the simulated results with the clinical data collected by Axelsson et al. (2010). With permission from the authors and the published journal we present details and results of the study below. The comparison will be in Section “Discussion.”

#### Description of the clinical study

One-hundred-twenty-six patients participated in the evaluation, 64 patients received mechanical chest compressions and 62 patients received manual chest compressions. Using a blinded protocol, 44 patients were randomized at the scene to have their legs elevated 35 cm during ongoing CPR, among them 23 patients received mechanical chest compressions and 21 patients received manual chest compressions. Partial Pressure of End-Tidal Carbon Dioxide (PETCO2), which has been shown a non-invasive method of detecting pulmonary blood flow and reflecting CO (Jin et al., 2000), was continuously monitored after the patient was intubated into tracheal. According to patient characteristics, there were no significant differences between patients who had their legs elevated vs. those that did not. More details of the clinical study are described in the reference (Axelsson et al., 2010).

#### Clinical results

There was a tendency toward a higher survival to hospital discharge among patients who had their legs elevated (7 vs. 1%; *p* = 0.12). Among all patients, time from CA to the start of CPR was 6 min and ROSC was obtained after 27 min. The measurement of PETCO2 was started 19 min after CA.

In Table 2 (reproduced from the original paper) the mean PETCO2 is compared between before and after PLR by 30, 60, and 90 s. Significant differences in the mean PETCO2 values were found among all patients (*n* = 44). A similar result was found if patients received manual chest compressions. We found no significant difference in the group receiving mechanical chest compressions. However, the mechanical group had a higher PETCO2 than the manual group in all measurements.

**Table 2:** **Comparison of mean PETCO2 values before and after passive leg raising in the referenced clinical study**.

	**All patients**	**Manual CPR**	**Lucas CPR**	P^*^
First measurement	*n* = 36	*n* = 16	*n* = 20	
30 s before PLR	2.69 ± 1.20	2.26 ± 0.88	3.04 ± 1.25	0.04
30 s after PLR	3.13 ± 1.41	2,90 ± 1.37	3.32 ± 1.45	0.38
After-before	0.44 ± 0.90	0.64 ± 0.88	0.28 ± 0.92	0.24
P^**^	0.006	0.01	0.19	
Second measurement	*n* = 33	*n* = 15	*n* = 18	
60 s before PLR	2.65 ± 1.15	2.30 ± 0.91	2.94 ± 1.27	0.11
60 s after PLR	3.10 ± 1.37	3.06 ± 1.49	3.14 ± 1.31	0.86
After-before	0.45 ± 0.87	0.76 ± 0.96	0.20 ± 0.73	0.07
P^**^	0.006	0.008	0.27	
Third measurement	*n* = 27	*n* = 13	*n* = 14	
90 s before PLR	2.56 ± 1.11	2.27 ± 0.93	2.84 ± 1.22	0.19
90 s after PLR	2.99 ± 1.13	2.81 ± 1.24	3.15 ± 1.25	0.48
After-before	0.43 ± 0.63	0.54 ± 0.48	0.32 ± 0.74	0.37
P^**^	0.002	0.002	0.13	

*Mean ± SD*.

P* *: p-value for the difference between manual CPR and Lucas CPR*.

P** *: p-value for the difference after-before PLR*.

## Results

**Figure 3:**
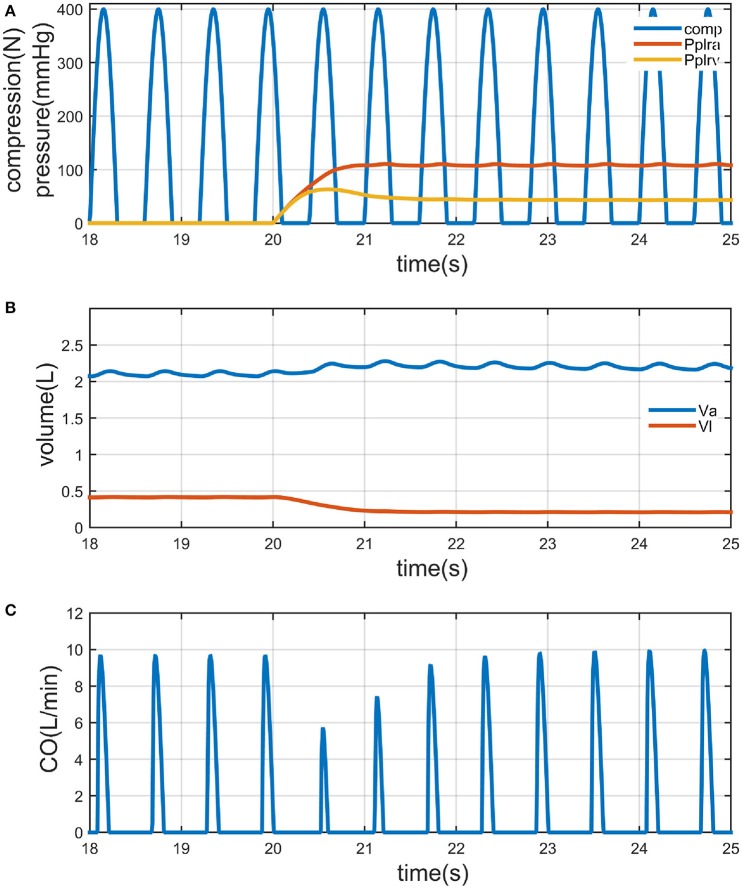
**Illustration of chest compression only CPR (CO-CPR) with PLR by 90° (PLR), when thoracic pump factor (TPF) is 0.75**. Legs are lifted since the twentieth second. **(A)** Curve of compression force to the thorax (“comp,” it is a non-overlapping half-sinusoid waveform with a 50% duty cycle, frequency: 100/min, amplitude: 400 N); and the curve of arterial pressure of legs led by PLR (“P_plra_,” unit is mmHg); and the curve of venous pressure of legs leg by PLR, (“P_plrv_,” unit is mmHg). **(B)** Curve of volume in abdomen (“V_a_,” unit is L), and curve of volume in legs (“V_l_,” unit is L). **(C)** Curve of cardiac output (L/min).

We first referred the value of TPF, for example, to 0.75 [in reference (Babbs, 2005), the blood in adult human can be expelled predominantly by thoracic pump mechanism], to show how PLR works on the circulation. Results are illustrated in Figure 3. Passive leg raising (by 90°) is performed since 20 s. The external compression force to the chest is the waveform of frequency 100/min, amplitude 400 N, non-overlapping half-sinusoids with a 50% duty cycle (Figure 3A). P_plra_, P_plrv_ is the pressure to the femoral arteries and veins arisen from the gravitational potential; the mean value of P_plra_ is 109 mmHg, the mean value of P_plrv_ is 43 mmHg. The volume in legs (V_l_) is decreased from 415 to 210 ml, 205 ml (49% of the volume) flows to the upper body; the volume in abdomen (V_a_) is increased from 2102 to 2186 ml, 84 ml stayed in the abdomen; the rest volume flows into the other compartments in the upper body (Figure 3B). At last the mean value of CO is increased by 9% (from 1.30 to 1.42 L/min) (Figure 3C).

**Figure 4:**
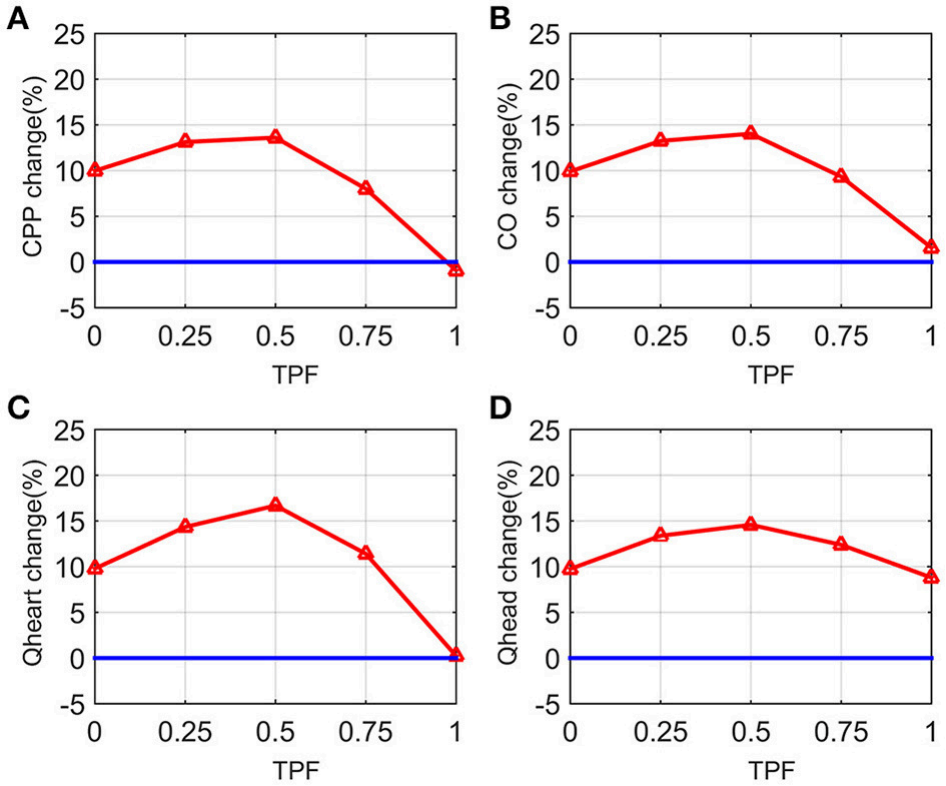
**Curves of changes in CPP, cardiac output (CO), blood flow to the heart (Q_**heart**_), blood flow to the brain (Q_**head**_) from chest compression only CPR (CO-CPR) to CO-CPR with PLR by 90° (PLR) when thoracic pump factor (TPF) is from 0 to 1**. CPP-change (%) = $\frac{\text{CP}{\text{P}}_{\text{with}\;\text{PLR}}-\text{CP}{\text{P}}_{\text{co}-\text{cpr}}}{\text{CP}{\text{P}}_{\text{co}-\text{cpr}}}\text{*}100\%$, CPP_co−cpr_ stands for CPP of chest compression only CPR (CO-CPR), CPP_with PLR_ stands for CPP of CO-CPR with PLR, CO-change (%), Q_heart_-change (%), and Q_head_-change (%) are calculated likewise. **(A)** Curve of CPP-change (%) when TPF is from 0 to 1. **(B)** Curve of CO-change (%) when TPF is from 0 to 1. **(C)** Curve of Q_heart_-change (%) when TPF is from 0 to 1. **(D)** Curve of Q_head_-change (%) when TPF is from 0 to 1.

Next, we simulated the change in CPP, CO, Q_heart_, and Q_head_ with PLR (by 90°), under different situations by varying TPF from 0 (pure cardiac pump, such as in the case of open chest or an extremely thin thorax) to 1 (pure thoracic pump, such as in the case of emphysema or an extremely thick and deep thorax). Results are presented in Figure 4. It is found in general PLR can accomplish better CPP, CO, Q_heart_, and Q_head_, except when TPF is 1. When TPF is reaching 1, all the compartments in the chest undergo almost the same pressure, CPP, CO, and Q_heart_ have no increase, while Q_head_ is increased by 9%; when TPF is around 0.5, the increase of CPP, CO, Q_heart_, and Q_head_ reach their maximum values. Q_head_ is increased by PLR regardless of the value of TPF.

**Figure 5:**
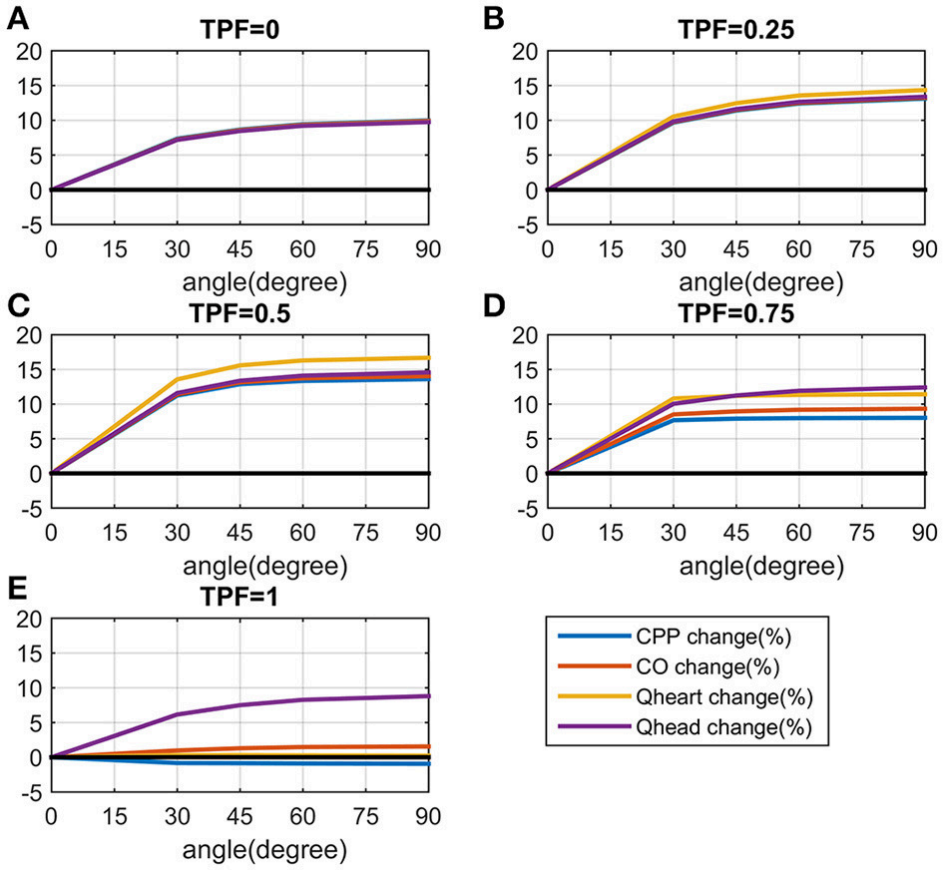
**Curves of changes in CPP, cardiac output (CO), blood flow to the heart (Q_**heart**_), blood flow to the brain (Q_**head**_) from chest compression only CPR (CO-CPR) to CO-CPR with legs are lifted to different degrees when thoracic pump factor (TPF) is 0, 0.25, 0.5, 0.75, 1**. CPP-change (%) = $\frac{\text{CP}{\text{P}}_{\text{with}\;\text{PLR}}-\text{CP}{\text{P}}_{\text{co}-\text{cpr}}}{\text{CP}{\text{P}}_{\text{co}-\text{cpr}}}\text{*}100\%$, CPP_co−cpr_ stands for CPP of chest compression only CPR (CO-CPR), CPP_with PLR_ stands for CPP of CO-CPR with PLR. CO-change (%), Q_heart_-change (%) and Q_head_-change (%) are calculated likewise. **(A)** The curves of CPP-change (%), CO-change (%), Q_heart_-change (%) and Q_head_-change (%) when TPF is 0. **(B)** The curves of CPP-change (%), CO-change (%), Q_heart_-change (%) and Q_head_-change (%) when TPF is 0.25. **(C)** The curves of CPP-change (%), CO-change (%), Q_heart_-change (%) and Q_head_-change (%) when TPF is 0.5. **(D)** The curves of CPP-change (%), CO-change (%), Q_heart_-change (%) and Q_head_-change (%) when TPF is 0.75. **(E)** The curves of CPP-change (%), CO-change (%), Q_heart_-change (%) and Q_head_-change (%) when TPF is 1.

The third we simulated the impact of the height to which the leg are lifted on these parameters. Results are presented in Figure 5. The most striking figure is the curves when TPF is 1. Obviously the parameters are not quite influenced by the height of the legs, except Q_head._ For other values of TPF, the parameters all increased with the angle of leg raising. However, it is also found that the rate of increase becomes much less when the angle is large. For example, when TPF is 0.75, the values of the parameters increase sharply when the angle is increased from 0 to 30°, but have much smaller changes when the angle is varied from 30° onwards.

**Figure 6:**
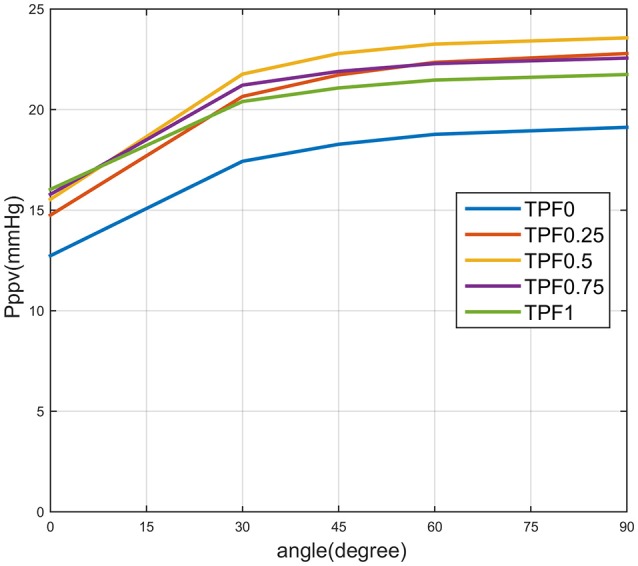
**Curves of pressure of peripheral pulmonary veins (Pppv) when angle is from 0 to 90°**. TPF0 stands for thoracic pump factor is 0, TPF0.25, TPF0.5, TPF0.75, and TPF1 are defined likewise.

Figure 6 shows the curves of peripheral pulmonary venous pressure (P_ppv_) under different values of TPF and different heights of leg raising. We found that peripheral pulmonary venous pressure, which has the similar value to pulmonary capillary pressure, is always lower than plasma colloid osmotic pressure [around 25–30 mmHg (Guyton et al., 1959)], it means CO-CPR with PLR did not cause pulmonary oedema (Zhang and Karemaker, 2012). PLR is safe for the lungs.

## Discussion

### *In silico* study

In this study we simulated the hemodynamic effect of CPR with PLR. According to the results it is found that in general PLR could accomplish increased CO, Q_heart_, and Q_head_. In the extreme case when pure thoracic pump mechanism works, CO, and Q_heart_ have no increase, but we still found Q_head_ is increased by 9%.

All along with the results, we found the least optimistic situation is when pure thoracic pump works (with an emphysema or extremely thick thorax). We gave Table 3 to compare the results of PLR under different situations, i.e., when pure cardiac pump, half cardiac/half thoracic pump, or pure thoracic pump mechanism works.

**Table 3:** **Comparison of cardiac output (CO), blood flow to heart (Q_**heart**_), blood flow to brain (Q_**head**_) when thoracic pump factor (TPF) is 0, 0.5, 1**.

	**TPF** = **0**			**TPF** = **0.5**			**TPF** = **1**		
	**Before**	**After**	**Increase**	**Before**	**After**	**Increase**	**Before**	**After**	**Increase**
	**PLR**	**PLR**	**by (%)**	**PLR**	**PLR**	**by (%)**	**PLR**	**PLR**	**by (%)**
CO	2.0	2.2	10	1.5	1.7	15	1.1	1.1	0
Q_heart_	1.9	2.1	10	1.4	1.6	17	0.8	0.8	0
Q_head_	6.9	7.6	10	5.8	6.6	14	4.8	5.2	9

Pure cardiac pump mechanism accomplished the highest absolute values for CO, Q_heart_, and Q_head_; half cardiac/half thoracic pump mechanism accomplished the largest increase in percentage change in the parameters with PLR; pure thoracic pump mechanism accomplished only the increase in Q_head_ with PLR. With pure cardiac pump mechanism the heart is massaged effectively, it is not surprised that it can accomplish the highest CO, Q_heart_, and Q_head_; as CO and Q_heart_ relied on how much is the venous return. With the high pressure to all the compartments in the chest when pure thoracic pump mechanism works, the volume from legs is hard to be transferred into the chest, it stayed in the abdomen. The simulated results told there were 101 ml more stayed in inferior vena cava when pure thoracic pump mechanism worked, while 58 ml when half cardiac/half thoracic pump mechanism, 24 ml when pure thoracic pump mechanism. However, Q_head_ does not only rely on CO, but also the systemic resistance of the lower body. Passive leg raising increased the height and thus the systemic resistance of the legs, and therefore there is less blood flow to the lower body, and more blood flow to the upper body. So with pure thoracic pump, even though there is no increase in CO, there is an increase in Q_head_ (by 9%).

In general PLR can increase the flow to the neck and brain (Q_head_) by 9–15% when TPF is varied from 0 to 1, which is important to increase cerebral perfusion. If TPF is <1 PLR can increase cardiac output, coronary and cerebral blood flow.

### Comparison with clinical results

In Section “Methods and Materials,” we gave a short description of a clinical study and results. In this section we compared the results of the simulation and the clinical results.

It has been verified that PETCO2 is positively correlated to CO (Von Planta et al., 1989) and PETCO2 is a non-invasive measurement of CO and ROSC (Asplin and White, 1995; Levine et al., 1997). In another study (Lewis et al., 1992). Some studies even quantified this correlation as Equation (8) (Shibutani et al., 1994; Maslow et al., 2001).

(8) $$\begin{array}{l} \text{y}=0.33\text{x}+0.13 \end{array}$$

Where x is the percent decreases (%) in cardiac output y is the percent decrease (%) in PETCO2.

In this model we simulated standard chest compression CPR in the same quality over time, it is therefore more similar to Lucas CPR group in Table 2, than manual CPR group. We compared the mean values of clinical results with the model results. Although there is no significant difference between before PLR and after PLR in mechanical CPR group, the mean values of PETCO2 are all higher after PLR. If we apply Equation (8) to the results found in Table 2, we can obtain the corresponding changes in CO for the clinical results. According to the clinical results, PLR generally increased PETCO2 by 0.20–0.32 kPa (mean values, about 7–11% increase, in the mechanical compression group), which is equivalently to having CO increased by about 20–30% with PLR. In the model simulation CO was increased by 14% the most (See in Figure 4), PLR has proved effective in improving CO in both studies.

In our model simulation results, we found that when TPF is reaching 1, the increases in CPP, CO, Q_heart_ would approach zero (refer in Figure 4E). If TPF of the patients is close to 1, it is possible that no significant increase in CO can be measured in the clinical study with PLR.

An important effect of PLR we found in our study is that it improves perfusion to the brain, regardless of the value of TPF (refer to Figures 3, 4). This could reduce the rate of brain injuries, which is a very important issue in CPR, specifically the patients who survived from CPR.

### Passive leg raising under cardiac and/or thoracic pump mechanism

How exactly does thoracic pump mechanism work in human body during CPR? This brings us back to the old debate: whether the thoracic- or cardiac-pump mechanism works during CPR performance. This study does not aim to argue in favor of any theory. According to “Current Concepts in Cardiopulmonary Resuscitation” (Torres and White, 1997), potentially, both the cardiac-pump and the thoracic-pump mechanisms may contribute to blood flow during CPR. The predominant mechanism may depend on anatomic or pathophysiological variations in a patient receiving CPR. We understand this as “individual differences.” A deep thorax is more related to the thoracic-pump effect, while a flat thorax is more related to the cardiac-pump effect. From the results, CPP, CO, and coronary perfusion could vary in different individuals, which could lead to varying results. This reminds us that, since 1992, CPR guidelines have no longer recommended PLR as an adjunct to CPR, possibly because it is not easy to obtain clear and significant evidence.

One important finding in this study is that when TPF is 1, we observed an increase in cerebral perfusion even when cardiac output has no increase, when PLR is applied. This can be explained as follows. Cardiac output depends on venous return. When TPF is 1, all the compartments in the chest undertake the same high compression pressure, the increased volume caused by PLR cannot get to the left ventricle easily. Therefore, there is no increase in cardiac output. On the other hand, cerebral perfusion depends on both cardiac output and the volume flow to the lower body. When PLR is applied, even though cardiac output is unchanged, leg raising reduced the volume flow to the lower body. Therefore, we can still observe an increase in cerebral perfusion.

When the legs were lifted from 0 to 30°, around 36% of the volume (150 ml) in legs flowed back to the central circulation. When the legs were lifted from 30 to 90°, 14% more volume (from 150 to 204 ml) in legs flowed back into the central circulation. Obviously further increase in the height of leg raising did not bring so much increase in the volume transferred. Therefore, it is not necessary for the legs to be lifted too high.

### Limitation

The model does not include the respiratory system and the effects of oxygenation by mouth-to-mouth breathing, PETCO2 cannot be simulated directly from the model. This computer model attempts to simulate the effects of PLR for different individuals, with a different thoracic-pump/cardiac-pump ratio. However, individuals can differ in more aspects: height, weight, age, disease history, and so on. To keep the model practical the supposed linearity of many anatomic and physiological systems is simplified, differences between model and outcome must exist in practice.

### Conclusion

In summary, the simulation results indicate that PLR increases the cerebral flow, in all situations. The increase in cerebral flow can improve brain perfusion, and thus reduce the chance of brain injury, which is important for the patients' quality of life if they survive. This study suggests that cerebral perfusion when PLR is applied should be measured in future clinical studies, in order to fully understand the effect of PLR, and to validate the results obtained from our simulation.

## Author contributions

YZ and CA designed the work, YZ was responsible for mathematical model developing, YZ, YC were responsible for analysis on results of modeling and simulation. CA, MJ were responsible for analysis on results of the referenced clinical study.

### Conflict of interest statement

The authors declare that the research was conducted in the absence of any commercial or financial relationships that could be construed as a potential conflict of interest.
